# Urinary Metabolites of Organophosphate Flame Retardants: Temporal Variability and Correlations with House Dust Concentrations

**DOI:** 10.1289/ehp.1205907

**Published:** 2013-03-05

**Authors:** John D. Meeker, Ellen M. Cooper, Heather M. Stapleton, Russ Hauser

**Affiliations:** 1 Department of Environmental Health Sciences, University of Michigan School of Public Health, Ann Arbor, Michigan, USA; 2 Nicholas School of the Environment, Duke University, Durham, North Carolina, USA; 3 Department of Environmental Health, Harvard School of Public Health, Boston, Massachusetts, USA; 4 Vincent Memorial Obstetrics and Gynecology Service, Massachusetts General Hospital, Boston, Massachusetts, USA

**Keywords:** biomarker, epidemiology, exposure, human, TDCPP, triphenyl phosphate

## Abstract

Background: A reduction in the use of polybrominated diphenyl ethers (PBDEs) because of human health concerns may result in an increased use of and human exposure to organophosphate flame retardants (OPFRs). Human exposure and health studies of OPFRs are lacking.

Objectives: We sought to define the degree of temporal variability in urinary OPFR metabolites in order to inform epidemiologic study design, and to explore a potential primary source of exposure by examining the relationship between OPFRs in house dust and their metabolites in urine.

Methods: Nine repeated urine samples were collected from 7 men over the course of 3 months and analyzed for bis(1,3-dichloro-2-propyl) phosphate (BDCPP) and diphenyl phosphate (DPP), metabolites of the OPFRs tris(1,3-dichloro-2-propyl) phosphate (TDCPP) and triphenyl phosphate (TPP), respectively. Intraclass correlation coefficients (ICCs) were calculated to characterize temporal reliability. Paired house dust and urine samples were collected from 45 men.

Results: BDCPP was detected in 91% of urine samples, and DPP in 96%. Urinary BDCPP showed moderate-to-strong temporal reliability (ICC range, 0.55–0.72). ICCs for DPP were lower, but moderately reliable (range, 0.35–0.51). There was a weak [Spearman *r* (*r*_S_) = 0.31] but significant (*p* = 0.03) correlation between urinary BDCPP and TDCPP concentrations in house dust that strengthened when nondetects (*r*_S_ = 0.47) were excluded. There was no correlation between uncorrected DPP and TPP measured in house dust (*r*_S_ < 0.1).

Conclusions: Household dust may be an important source of exposure to TDCPP but not TPP. Urinary concentrations of BDCPP and DPP were moderately to highly reliable within individuals over 3 months.

Flame retardant chemicals are commonly added to consumer products and raw materials to delay combustion and meet fire safety standards. Because many flame retardants are additive rather than chemically bonded with the material, over time they can leach out of the treated materials, which may result in human exposure through various pathways and routes. This includes ingestion, inhalation, and dermal contact with household dust, among other sources (Johnson et al. 2010; Stapleton et al. 2012; Watkins et al. 2012). Flame retardants such as polybrominated diphenyl ethers (PBDEs) and others have received much attention lately, as widespread human exposure has been documented and concerns for health risks have increased based on recent reports from human and animal research (Birnbaum and Bergman 2010; DiGangi et al. 2010; Shaw et al. 2010). This has led to bans or voluntary phase-outs of several commercial PBDE mixtures from the marketplace in many countries. However, with the halted or reduced use of PBDEs throughout the world, there are new concerns regarding human exposure and possible health effects associated with the alternative flame retardants that may be increasingly used to replace PBDEs.

Of a number of available alternatives to PBDEs, the use of organophosphate flame retardants (OPFRs), such as the triesters tris(1,3,-dichloro-2-propyl) phosphate (TDCPP) and triphenyl phosphate (TPP), are currently widespread and expected to increase (U.S. Environmental Protection Agency 2005). TDCPP and TPP are used as additive flame retardants in polymers, resins, latexes, and foams; TPP is also used as a plasticizer and lubricant and in hydraulic fluids (van der Veen and de Boer 2012). The United States is likely the largest user of TDCPP and TPP, consuming between 4,500 and 22,700 metric tons of each chemical annually (van der Veen and de Boer 2012). However, data on human exposure and potential health risks related to OPFRs are lacking. We recently detected TDCPP and TPP in 96% and 98% of U.S. house dust samples analyzed, respectively, with concentrations similar to or greater than those measured for PBDEs in the same samples (Stapleton et al. 2009). TDCPP was detected in 15 of 26 samples of polyurethane foam collected from different types of residential furniture, and it was the most common flame retardant detected in infant products, in a recent survey (Stapleton et al. 2011). It was detected in 36% of 102 samples that were analyzed, at concentrations that accounted for approximately 4–5% of the foam by weight.

Limited animal studies suggest that certain OPFRs may be carcinogenic, neurotoxic, and/or reproductive toxicants (Dishaw et al. 2011; van der Veen and de Boer 2012). As far as we are aware, our previous study reporting relationships of TDCPP and TPP in house dust with hormone levels and semen quality in men remains the only human study on these OPFRs to date (Meeker and Stapleton 2010). More experimental animal and human epidemiology research is needed to determine potential health risks resulting from exposure to OPFRs.

Adequate assessment of exposure is a vital component for reducing measurement error in epidemiologic studies. Measurement error can be detrimental to the ability to detect potential associations between an exposure and health outcome with adequate power, accuracy, and precision. For flame retardants, measuring chemical concentrations in dust or relevant biomarkers in various biological specimens are two options for assessing exposure in epidemiologic studies. Measurements of the parent compounds and/or metabolites can prove to be useful biomarkers of exposure. For OPFRs, metabolism can be quite rapid. Previous studies in rodents demonstrated that TDCPP and TPP were rapidly metabolized, and the primary metabolites identified were dialkyl metabolites, bis(1,3-dichloro-2-propyl) phosphate (BDCPP), and diphenyl phosphate (DPP), respectively (Lynn et al 1981; Nomeir et al 1981; Sasaki et al 1984). Furthermore, a recent study investigating the *in vitro* metabolism of these same two OPFRs in human liver microsomes demonstrated that the primary metabolites in humans were also likely BDCPP and DPP (Cooper and Stapleton 2012). We recently developed a method to extract and measure BDCPP and DPP metabolites in human urine (Cooper et al. 2011). In that study, nine randomly collected urine samples from nonoccupationally exposed adults all had detectable concentrations of both metabolites. Urinary concentrations of BDCPP ranged from 0.04 to 1.66 ng/mL, and DPP concentrations ranged from 0.15 to 1.07 ng/mL. However, the method has yet to be applied to a larger study. Furthermore, because TDCPP and TPP are metabolized to BDCPP, DPP, and other metabolites and rapidly excreted, more information is needed on how variable urinary metabolite concentrations are within individuals over time. If TDCPP and TPP are persistent in house dust, and house dust is the primary source of exposure, urinary metabolite concentrations may be relatively stable over time despite their short biological half-lives if individual time–activity patterns are consistent.

The goals of the present study were to *a*) characterize the distribution of urinary BDCPP and DPP in a population of U.S. men from an ongoing study; *b*) define the degree of temporal variability in these OPFR metabolites in order to inform exposure assessment efforts in epidemiologic studies; and *c*) determine the relationship between OPFRs in house dust and their metabolites in urine as a first step in identifying possible sources, pathways, and routes important for human exposure. Identification of how exposure takes place is a critical step for risk management through effective exposure reduction strategies.

## Methods

*Study participants*. The present study was conducted on a subset of men participating in an ongoing study of environmental factors in reproductive health. Details of subject recruitment into the parent study have been previously described (Meeker et al. 2005, 2007). Briefly, men between 18 and 54 years of age were recruited from the Vincent Memorial Andrology laboratory at Massachusetts General Hospital and invited to participate in the study. Applicable requirements involving human subjects were followed; institutional review board approval was obtained from each participating institution and all subjects provided written informed consent. Approximately 65% of eligible men agreed to participate. Exclusionary criteria included prior vasectomy or current use of exogenous hormones.

We previously analyzed TDCPP and TPP concentrations in house dust collected from the homes of 50 randomly selected men participating in the parent study who were recruited between years 2002 and 2007 (Meeker and Stapleton 2010). For the present study, 45 of the 50 men had archived urine samples with enough volume to measure OPFR metabolites to evaluate agreement between the concentrations of the parent chemical in house dust with its metabolite in urine. Dust and urine samples from the same men were typically collected within a month of one another, with a median of 14 days. A urine sample was collected at the clinic visit upon study recruitment, and the participants were given prepaid shipping material and detailed instructions to send the vacuum bag that was in their home vacuum cleaner at the time to the research team for analysis.

The parent study also included a variability substudy, where a subset of men were recruited to provide 9 repeated urine samples over the course of 3 months (the approximate duration of spermatogenesis) in order to determine temporal variability of various exposure biomarkers. A urine sample was collected on each of 3 repeated days in three separate cycles that were 1 or 2 months apart; upon enrollment into the variability study, men provided urine samples from days 1, 2, 3, 30, 31, 32, 91, 92, and 93. Seven of these men from the variability substudy were included in the present analysis. One of the seven men had 1 missing repeat urine sample, and 1 sample from a second man had insufficient volume for extraction. Thus, a total of 61 urine samples were analyzed from the variability substudy.

*Urine analysis*. Urine samples, which were collected in glass vials [either a 30 mL Qorpak vial (Qorpak, a division of Berlin Packaging, Bridgeville, PA) or a 25 mL Wheaton vial (Wheaton Industries, Millville, NJ)] and stored at –80°C, were analyzed for BDCPP and DPP as previously described (Cooper et al. 2011). Briefly, BDCPP and DPP were measured in urine samples using mixed-mode anion exchange solid phase extraction and a mass-labeled internal standard [deuterated (d10)-BDCPP and d10-DPP] with analysis by atmospheric pressure chemical ionization liquid chromatography–tandem mass spectrometry. For quality assurance purposes, we evaluated BDCPP and DPP levels in laboratory blanks (deionized water; *n* = 6) and ran 12 samples in duplicate. Recoveries of the internal quantification standards, d10-BDCPP and d10-DPP, averaged 88 ± 16% and 89 ± 15%, respectively. BDCPP and DPP were present at very low levels in laboratory blanks and averaged 0.06 ± 0.05 ng and 0.14 ± 0.09 ng, respectively. The method detection limit was calculated using three times the SD of the blanks normalized to the urine volume extracted, which was 32.7 pg/mL and 55.3 pg/mL, for BDCPP and DPP respectively. Specific gravity (SG) was measured in each urine sample before analysis using a digital handheld refractometer.

*Dust analysis*. Dust from participants’ household vacuum bags was sieved (150 μm screen) and analyzed for TDCPP and TPP as described in our previous report (Meeker and Stapleton 2010). Briefly, 0.3–0.5 g of sieved dust was extracted in stainless steel cells with a 50:50 vol:vol mixture of dichloromethane:hexane using pressurized fluid extraction (ASE 300; Dionex Inc., Sunnyvale, CA). Final extracts were reduced in volume to approximately 1.0 mL using an automated nitrogen evaporation system (Turbo Vap II; Zymark Inc., Hopkinton, MA) and were purified by elution through a glass column containing 4.0 g of 6% deactivated alumina. The final extract was then reduced in volume to 0.5 mL before gas chromatography–mass spectrometry (GC/MS) analysis. Samples were analyzed using GC/MS operated in either electron impact mode (GC/EI-MS) for TPP, or electron capture negative ionization mode (GC/ECNI-MS) for TDCPP. TDCPP was quantified by monitoring *m/z* 319 and 317, and TPP was quantified by monitoring *m/z* 326 and 325. As further confirmation, all ion ratios were monitored and were within 20% of their expected values as compared with authentic standards.

As part of our quality assurance criteria, we examined levels of these specific analytes in laboratory blanks (sodium sulfate and solvent only, *n* = 4), replicate samples (*n* = 3), and in matrix spikes (*n* = 3). Sample measurements were blank corrected by subtracting the average level measured in the laboratory blanks. Blank levels for TDCPP and TPP were 11.7 ± 6.6 and 15.7 ± 11.9 ng, respectively. Method detection limits were calculated as three times the SD of the blank levels. Matrix spikes were prepared by adding between 25 and 100 ng of TDCPP and TPP to the ASE cells [stainless steel cells used on the ASE 300 (Dionex Inc.)] filled with sodium sulfate powder. Matrix spikes were extracted using the same method used for dust and examined for percent recovery using 50 ng of ^13^C chlorinated diphenyl ether 141 as an internal standard. Recoveries averaged 86 ± 7% and 89 ± 2% for TDCPP and TPP, respectively.

*Statistical analysis*. Statistical analysis was carried out using SAS version 9.2 (SAS Institute Inc., Cary, NC). Distributions of urinary BDCPP and DPP concentrations, along with dust concentrations of TDCPP and TPP, were tabulated. Graphs were constructed to visually and qualitatively compare urinary metabolite concentrations over time within and between subjects. To quantitatively assess between- and within-person variability in urinary BDCPP and DPP concentrations, intraclass correlation coefficients (ICCs) and their 95% confidence intervals (CIs) were calculated using mixed random effects models in SAS PROC MIXED (Hankinson et al. 1995; Rosner 2000). ICC is a measure of the reliability of repeated measures over time, defined as the ratio of between-subject variance and total variance. ICC ranges from zero to one, with values near zero indicating poor reliability and values near one indicating high reliability (Rosner 2000).

In addition to calculating ICCs, we also conducted a sensitivity analysis to evaluate how well OPFR metabolite concentrations from a single urine sample (the “predicted” or “surrogate” value) could correctly classify men into the highest exposure category based on their 3-month geometric mean (GM) concentration (the “observed” or “true” value) calculated from the nine repeated samples (Hauser et al. 2004; Mahalingaiah et al. 2008; Meeker et al. 2005). In this analysis, the seven men in the variability study were first categorized into exposure groups for both BDCPP and DPP based on their 3-month GM concentrations using three different cutoffs: *a*) men with the four highest 3-month exposure levels (> 43rd percentile), *b*) men with the three highest 3-month exposure levels (> 57th percentile), or *c*) men with the two highest 3-month exposure levels (> 71st percentile). Men were then ranked by exposure for each individual time point (e.g., day 1, 2, 3, 30) based on the concentrations measured in that individual sample. A contingency table was then constructed for each time point comparing predicted and observed categories. Contingency tables from all 9 time points were then combined into a single table, where sensitivity, specificity, positive predictive value, and negative predictive value were calculated for each metabolite and for each of the three category cutoff schemes.

Finally, scatter plots were constructed to assess the relationship between dust and urine metabolite concentrations. Spearman rank correlations (*r*_S_) were then calculated to evaluate the relationships between concentrations of TDCPP and TPP in house dust and concentrations of their metabolites in urine from the same men from their clinic visit at study entry. For urinary metabolites, all analyses were conducted for both uncorrected concentrations and concentrations corrected for specific gravity using the following formula:

*P_c_* = *P* × [(1.024–1)/(*SG*–1)], [1]

where *P_c_* is the SG-corrected metabolite concentration (in nanograms per milliliter), *P* is the observed metabolite concentration (in nanograms per milliliter), and *SG* is the specific gravity of the urine sample (Boeniger et al. 1993).

## Results

Overall, of the 106 samples we analyzed for the two OPFR metabolites, BDCPP was detected in 96 samples (91%), and DPP was detected in 102 samples (96%). Table 1 shows the distribution of BDCPP and DPP concentrations in urine, as well TDCPP and TPP in dust, from the 45 men who contributed a single sample of both matrices. Distributions of all analytes spanned several orders of magnitude and were highly right-skewed. BDCPP and DPP in urine were moderately correlated with one another (*r*_S_ = 0.54; *p* < 0.05).

**Table 1:** Distribution (GM and selected percentiles) of urinary DBCPP and DPP concentrations (ng/mL), and paired TDCPP and TPP concentrations (ng/g) in house dust (*n* = 45).

Variable	Percent NDa	GM	Percentile						Maximum
			10th	25th	50th	75th	90th	95th	
Urine (ng/mL)
BDCPP	9	0.13	ND	0.03	0.12	0.27	0.89	1.89	25.0
DPP	4	0.31	0.07	0.14	0.27	0.75	1.64	2.65	9.84
Dust (ng/g)
TDCPP	4	1,580	181	891	1,620	2,450	14,390	47,000	56,080
TPP	2	6,836	600	2,925	6,000	9,170	180,450	319,820	1,798,000
ND, nondetect. aRate based on all 106 urine samples analyzed.									

**Figure 1:**
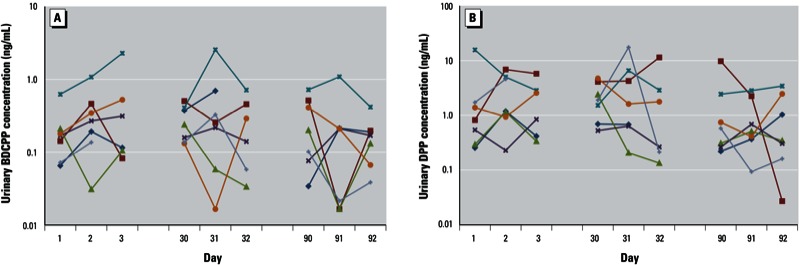
Concentrations (ng/mL) in urine from repeated samples collected from seven men over a 3-month period. (*A*) BDCPP; (*B*) DPP. Colors represent individual men.

Figure 1 shows concentrations of BDCPP and DPP measured in nine repeated samples collected from seven men who participated in the variability substudy over the course of 3 months (two men were missing a sample at one of the time points; *n* = 61). For BDCPP (Figure 1A), one man had noticeably higher concentrations for 8 of the 9 sample days, whereas there appeared to be more overlap in DPP concentrations between men over time (Figure 1B). Table 2 displays ICCs for these data. BDCPP showed moderate-to-strong temporal reliability depending on whether concentrations were SG-corrected and/or nondetect samples were excluded (ICC range, 0.55–0.72) (Rosner 2000). ICCs for DPP were lower but moderately reliable (range, 0.35–0.51).

**Table 2:** ICCs (95% CIs) for uncorrected and SG-corrected urinary BDCPP and DPP concentrations.

Urinary metabolite	All samplesa		Excluding nondetectsb	
	Uncorrected	SG-Corrected	Uncorrected	SG-Corrected
BDCPP	0.62 (0.40, 0.80)	0.55 (0.31, 0.77)	0.70 (0.50, 0.84)	0.72 (0.53, 0.85)
DPP	0.36 (0.18, 0.60)	0.35 (0.17, 0.59)	0.51 (0.32, 0.70)	0.50 (0.29, 0.70)
an = 106 samples from 51 men. bn = 102 samples from 48 men for DPP, n = 96 samples from 44 men for BDCPP.				

Results from our sensitivity analysis for the ability of a single sample to correctly classify men as being more highly exposed based on their 3-month GMs (not SG corrected) are shown in Table 3. Both metabolites showed high sensitivities (range, 0.70–0.86), with DPP showing somewhat greater agreement and more consistency between classification approaches compared with BDCPP. Results were similar when calculating sensitivity, specificity, positive predictive value, and negative predictive value using SG-corrected concentrations (data not shown).

**Table 3:** Sensitivity and specificity analysis for the ability of a single urine measurement to correctly classify a high exposure group among 7 men according to 3-month GM metabolite concentration.

Exposure classification	Sensitivity	Specificity	PPV	NPV
Highest 4 of 7
BDCPP (> 0.165 ng/mL)	0.75	0.64	0.75	0.64
DPP (> 0.545 ng/mL	0.86	0.65	0.77	0.77
Highest 3 of 7
BDCPP (> 0.175 ng/mL)	0.70	0.56	0.56	0.70
DPP (> 1.15 ng/mL)	0.81	0.79	0.76	0.84
Highest 2 of 7
BDCPP (> 0.190 ng/mL)	0.83	0.60	0.47	0.90
DPP (> 1.99 ng/mL)	0.83	0.86	0.71	0.93
Abbreviations: PPV, positive predictive value; NPV, negative predictive value. Concentrations not corrected for SG.				

**Figure 2:**
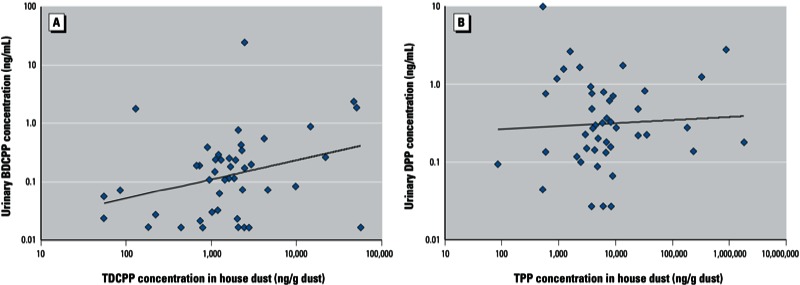
Relationship between house dust OPFR concentrations (ng/g of dust) and uncorrected urinary metabolite concentrations (ng/mL) for 45 study participants with paired samples. (*A*) TDCPP and BDCPP (*r_S_* = 0.31, *p* = 0.03). (*B*) TPP and DPP (*r_S_* = 0.04, *p* = 0.8).

Figure 2 displays scatter plots of the relationship between uncorrected urinary BDCPP and DPP concentrations and previously measured concentrations of TDCPP and TPP, respectively, in house dust (*n* = 45). Uncorrected urinary BDCPP was weakly (*r*_S_ = 0.31) but significantly (*p* = 0.03) correlated with TDCPP concentrations in house dust (Figure 2A). This correlation weakened when using SG-corrected BDCPP concentrations (*r_S_* = 0.25; *p* = 0.10). There was no correlation between uncorrected (*r_S_* = 0.04; *p* = 0.8) (Figure 2B) or SG-corrected (*r_S_* = 0.03; *p* = 0.8) DPP and TPP measured in house dust. When excluding seven nondetects (not shown), the correlation of TDCPP with BDCPP increased to 0.47 (*p* = 0.003). However, removing three nondetects did not improve the correlation between TPP and DPP (*r_S_* = 0.05). Finally, we calculated correlations when stratifying by time between collection of urine and dust samples, but correlations did not improve when limiting the analysis to samples collected within 2 weeks (*n* = 22) or within 1 week (*n* = 10) of one another.

## Discussion

BDCPP and DPP were detected in nearly all urine samples analyzed from a study of exposure to environmental chemicals and reproductive health among men from the Boston, Massachusetts, area. This confirms the previous findings from our method development study that found detectable levels of both DBCPP and DPP in all nine samples randomly collected from adults in North America (Cooper et al. 2011). In both that study and in the present analysis, a very broad range of concentrations spanning four orders of magnitude were found, suggesting exposure to OPFRs is widespread but may be highly variable among U.S. adults.

Although toxicokinetic information on TDCPP and TPP is limited, studies using rat and human liver microsomes suggest that TDCPP and TPP are metabolized rapidly to diesters (e.g., BDCPP and DPP, respectively) and other metabolites (Cooper and Stapleton 2012; Lynn et al. 1981; Nomeir et al. 1981; Sasaki et al. 1984). Thus, the half-lives of the parent compounds in blood, and of their metabolites in urine, are likely very short, on the order of several hours. The development of exposure biomarkers of OPFRs that can be measured in blood or urine is a significant step toward improving the existing data on human health risk in relation to these chemicals. However, as with other nonpersistent environmental contaminants, if a single urine sample is used to define individual exposure in an epidemiologic study, knowledge of its temporal variability is needed to determine whether the measure is reliable over a period of time commensurate with the development of the outcome measure(s) of interest in order to minimize exposure measurement error and misclassification. To address this, we calculated ICCs and sensitivity to determine the reliability of BDCPP and DPP over the course of 3 months.

In the ICC analysis, there was moderate reliability for DPP (ICC = 0.4–0.5), and moderate to strong reliability for BDCPP (ICC = 0.6–0.7). The ICCs for both metabolites were higher than other urinary biomarkers of nonpersistent chemicals that have been measured in overlapping groups of men from the same cohort (Hauser et al. 2004; Mahalingaiah et al. 2008; Meeker et al. 2005), especially for chemicals resulting from exposure primarily through diet. For example, there were considerably weaker ICCs in the ongoing study for bisphenol A (BPA; ICC = 0.2), mono-2-ethylhexyl phthalate (MEHP; ICC = 0.3), and 3,5,6-trichloro-2-pyridinol (TCPY, a metabolite of the organophosphate insecticides chlorpyrifos and chlorpyrifos-methyl; ICC = 0.2). Thus, the higher ICCs for OPFR metabolites may indicate direct and stable sources, such as indoor dust, may be significant contributors to exposure. For BDCPP, one man had notably higher concentrations on most sample days, suggesting a consistent source of exposure for that participant. This participant was also the only variability subject for whom we had measured OPFR dust concentrations to compare with urinary metabolites at study entry. He had one of the higher concentrations of both dust TDCPP and urinary BDCPP among the 45 men with paired dust and urine samples (14,391 ng/g TDCPP, 5th highest dust concentration of the 45 men with paired data; 0.89 ng/mL BDCPP; also 5th highest urinary concentration among the 45 men with paired data).

Both metabolites showed high sensitivities for a single urine sample to predict high exposure groups based on 3-month GM concentrations. For DPP, which did not perform as well in the ICC analysis compared with BDCPP, this may indicate that there may be significant within-person variability in concentrations over time, but that DPP concentrations among the most highly exposed individuals tend to remain among the highest relative to other individuals over the course of 3 months. However, it should be noted that the sensitivity analysis included samples from only seven men and that results may be unstable because of the relatively small sample size. Future studies should assess the temporal variability of these and other OPFR markers of exposure in a larger number of participants.

Dust in the home and in other environments has been shown to be a major source of exposure for other flame retardants such as PBDEs, with hand-to-mouth contact representing one of the primary exposure pathways (Johnson et al. 2010; Stapleton et al. 2012; Watkins et al. 2012). However, empirical data on potential sources, pathways, and routes of exposure to OPFRs are lacking. In our comparison of 45 paired house dust and urine samples, BDCPP concentrations in urine were moderately but significantly correlated with TDCPP concentrations in house dust. Thus, house dust may be an important source of exposure to TDCPP among adult men. The lack of a very strong correlation between house dust and urinary metabolite concentrations is not unexpected, given the rapid metabolism of TDCPP and moderate-to-strong temporal reliability in BDCPP concentrations. Other microenvironments containing TDCPP, such as workplaces, may also be significant contributors to TDCPP exposure (and, subsequently, urinary BDCPP concentrations) as suggested in a recent Belgian study that found that dust concentrations of TDCPP were much greater in certain work environments compared with house dust (Van den Eede et al. 2011). However, it should be noted that TDCPP concentrations in house dust from Belgium are likely significantly lower than those found in house dust from the United States. Future studies should be designed to further determine sources, pathways, and routes of exposure to OPFRs (e.g., vehicles, work, school, daycare), especially among children because they experience greater doses per body weight, typically have greater contact with house dust, and frequent hand-to-mouth contact and are at sensitive stages of rapid growth and development. Research is also needed on the variability of OPFR concentrations in dust samples over time within a given microenvironment, and whether OPFR metabolites are present in the environment and contribute to urinary concentrations.

We found no correlation between TPP in house dust and DPP in urine. This suggests that TPP in house dust may not be a primary source of exposure among adult men. As opposed to halogenated OPFRs such as TDCPP, which tend to be primarily used as flame retardants, nonderivatized alkyl phosphates such as TPP may be predominantly used as plasticizers and lubricants or in other applications (van der Veen and de Boer 2012). These different and diverse uses may result in sources and pathways of exposure that are quite unlike those for chemicals that are used primarily as flame retardants. In addition, TPP has a higher vapor pressure than TDCPP, so it is possible that inhalation of TPP in indoor air may play a more significant role in total exposure compared with TDCPP and other less volatile flame retardants. Other possibilities for the lack of correlation between TPP and DPP in our data include the potential for other chemicals to be metabolized to DPP, the potential for exposure to DPP directly if DPP is widely used in other applications (Makiguchi et al. 2011), or after environmental degradation of TPP to DPP and potential interindividual differences in exposure profiles, scenarios, or toxicokinetics. Differences in toxicokinetics between two persons with the same level of exposure could result in significant differences in the amount of a specific metabolite excreted in urine. Circumstances of sample collection may also contribute to the lack of correlation between TPP and DPP, and may have limited the strength of the correlation we calculated between TDCPP and BDCPP. For example, dust samples were obtained from participants’ vacuum bags as opposed to researcher-collected samples, and the time between collection of dust and urine samples varied between participants. However, limiting the analysis to samples collected closer together in time did not improve the strength of the correlation between dust OPFR concentrations and the measured metabolites in urine (not shown).

## Conclusions

Exposure to TDCPP and TPP among adult men in the United States is likely common. In the present study a high degree of variability between persons for concentrations of TDCPP and TPP in house dust and their metabolites in urine was observed, and urinary concentrations of BDCPP and DPP were moderately to highly reliable within individuals over the course of 3 months. Our data suggest that household dust may be an important source of exposure to TDCPP but not TPP, although more detailed research on the exposure sources, pathways and routes of exposure to these chemicals is needed. If the indoor environment at home is the primary source of exposure to OPFRs, measuring concentrations in house dust may be a desirable approach to assessing long-term exposure to these chemicals that are likely metabolized and excreted rapidly once entering the body. However, concentrations in dust from other microenvironments where people spend the most time may also need to be considered, which would increase the time and costs required and may become infeasible in large epidemiologic studies. On the other hand, if sources of OPFR exposure are multiple and varied, exposure biomarkers such as urinary metabolites that account for all routes of exposure may be the best approach. However, knowledge of their utility through a better understanding of toxicokinetics, temporal variability, and other parameters is needed.
